# ALW peptide ameliorates lupus nephritis in MRL/lpr mice

**DOI:** 10.1186/s13075-019-2038-0

**Published:** 2019-12-02

**Authors:** Huixia Wang, Mei Lu, Siyue Zhai, Kunyi Wu, Lingling Peng, Jie Yang, Yumin Xia

**Affiliations:** 1 Department of Dermatology, The Second Affiliated Hospital, School of Medicine, Xi’an Jiaotong University, Xi’an, 710004 China; 2 Core Research Laboratory, The Second Affiliated Hospital, School of Medicine, Xi’an Jiaotong University, Xi’an, China; 3 Department of Nephrology, Tangdu Hospital, The Fourth Military Medical University, Xi’an, 710032 China

**Keywords:** ALW peptide, Lupus nephritis, Anti-dsDNA IgG, Inflammation, Fibrosis

## Abstract

**Background:**

Lupus nephritis (LN) is a common and serious complication of systemic lupus erythematosus. Anti-double-stranded (ds) DNA immunoglobulin G (IgG) plays a pivotal role in the pathogenesis of LN. Currently, there are various therapies for patients with LN; however, most of them are associated with considerable side effects. We confirmed previously that ALW (ALWPPNLHAWVP), a 12-amino acid peptide, inhibited the binding of polyclonal anti-dsDNA antibodies to mesangial cells and isolated glomeruli in vitro. In this study, we further investigate whether the administration of ALW peptide decreases renal IgG deposition and relevant damage in MRL/lpr lupus-prone mice.

**Methods:**

Forty female MRL/lpr mice were randomly divided into four groups. The mice were intravenously injected with D-form ALW peptide (ALW group), scrambled peptide (PLP group), and normal saline (NaCl group) or were not treated (blank group). The IgG deposition, the histopathologic changes, and the expressions of profibrotic factors were analyzed in the kidney of MRL/lpr mice.

**Results:**

Compared with the other groups, glomerular deposition of IgG, IgG2a, IgG2b, and IgG3 was decreased in the ALW group. Moreover, ALW administration attenuated renal histopathologic changes in MRL/lpr mice, including mesangial proliferation and infiltration of inflammatory cells. Furthermore, the expressions of profibrotic cytokines, such as transforming growth factor-beta1 (TGF-β1) and platelet-derived growth factor B (PDGF-B), decreased in the serum and kidney tissue of ALW-treated mice.

**Conclusions:**

Our study demonstrated that ALW peptide ameliorates the murine model of LN, possibly through inhibiting renal IgG deposition and relevant tissue inflammation and fibrosis.

## Background

Systemic lupus erythematosus (SLE) is an autoimmune disease characterized by a large amount of autoantibodies and involvement of autoantibody-induced end-organ damage which is associated with significant morbidity and mortality [1]. Lupus nephritis (LN) is one of the most common and severe complications of SLE. There are many factors involved in the pathogenesis of LN. Anti-double-stranded (ds) DNA antibody plays a crucial role in renal injuries by indirect and direct binding with glomerular antigens [2–4]. The cross-reaction between anti-dsDNA immunoglobulin G (IgG) and renal antigens activates complement cascades, regulates gene expression, promotes cellular proliferation, and induces phenotypic changes of glomerular resident cells, leading to special damage in LN [5–7]. In addition, anti-dsDNA antibodies induce transdifferentiation of tubular epithelial myofibroblasts, increase the expressions of transforming growth factor-beta1 (TGF-β1), and increase synthesis of soluble fibronectin and collagen by proximal tubular epithelial cells, which are signs of renal fibrosis in LN [8, 9]. At present, cytotoxic agents and corticosteroids are standardized managements for patients with LN; however, they are associated with considerable side effects [10]. Immunosuppressive agents, particularly mycophenolate mofetil, are effective and well tolerated in the treatment of LN, whereas viral infection and diarrhea are frequently observed due to immune inhibition [11]. Therefore, finding feasible treatments with fewer unwanted outcomes is critical.

Previously, we found that the renal pathogenicity of anti-dsDNA IgGs (IgM, IgG1, IgG2a, IgG2b, and IgG3) was affected by the isotypes in a murine model [12, 13]. IgG2a and IgG3 had higher affinity for autoantigens in the kidney, followed by IgG1, IgG2b, and IgM [12]. DWEYS peptide (DWEYSVWLSN) is a DNA-mimic peptide screened by a mouse pathogenic monoclonal antibody (IgG2b, R4A clone). After intraperitoneal administration of D-form DWEYS peptide, renal IgG deposition was evidently depressed in a murine model [14]. FISLE-412, a peptide compound mimicking the molecular structure of DWEYS peptide, had much higher affinity to anti-dsDNA antibodies and highly prevented their pathogenic interaction with tissue antigens [15]. Interestingly, we identified a 12-amino acid peptide (ALWPPNLHAWVP [ALW]) that effectively inhibited the binding of all murine and human anti-dsDNA IgG subclasses to glomerular antigens [16]. Preincubation with the ALW peptide significantly decreased the reaction of the PL9-11 anti-DNA antibodies with DNA, laminin, mesangial cells, and isolated glomeruli in vitro [16]. Moreover, ALW peptide markedly inhibited the affinity of murine and human lupus sera to dsDNA and glomeruli in vitro [16]. Accordingly, through blocking the reactions of polyclonal anti-dsDNA antibodies to autoantigens in vivo, the ALW peptide (or its derivatives) may possibly be a helpful approach to depress the pathogenicity of anti-DNA antibodies.

Therefore, to further investigate the effect of ALW peptide on nephritis in vivo, we intravenously injected the D-form ALW peptide into MRL/lpr lupus-prone mice, a murine model of spontaneous lupus nephritis, and then observed the development of renal injury.

## Methods

### Peptides

Two 12-amino acid peptides (ALWPPNLHAWVP [ALW]; PLPHNPWVLAAW [PLP]) were synthetized by Sangon Biotech Company (China). The PLP peptide was a scrambled peptide with amino acid residues identical to ALW peptide. The first and last amino acids of both peptides are D-form, and the others are L-form. To study the pharmacokinetic characteristics of both peptides in vivo, 8-week-old male CD1 mice were administrated with ALW or PLP peptide at a single dose of 12.5 mg/kg. Plasma samples were collected at each time point before (0) and 0.083, 0.25, 0.5, 1, 2, 4, 8, and 24 h after administration. To assess the biodistribution of ALW peptide in vivo, tissues including the kidney, liver, and lung were collected at 0.083, 0.25, 0.5, 1, 2, and 4 h after administration. Then, the concentration of two peptides in mouse plasma and tissues were quantitatively determined by liquid chromatography-tandem mass spectrometry (LC-MS/MS). The pharmacokinetic parameters were calculated by WinNonlin 6.1 software according to the non-atrioventricular model.

### Mice

Seven-week-old female MRL/lpr mice were purchased from Shanghai Laboratory Animal Center (China) and housed under specific pathogen-free (SPF) conditions at the Medical Animal Center of Xi’an Jiaotong University. The mice were acclimatized in the facility for 2 weeks prior to the experiments.

For the in vivo study, 40 MRL/lpr mice were randomly divided into four groups. Mice in the ALW group (*n* = 12) received an intravenous injection of 100 μg/mL ALW peptide in saline every other day, mice in the PLP group (*n* = 12) were injected intravenously with 100 μg/mL PLP peptide in saline, mice in the NaCl group (*n* = 8) were given normal saline intravenously, and mice in the blank group (*n* = 8) were not treated. The dosages were decided in a preliminary experiment, showing that the concentration of anti-dsDNA IgG was about 34.74 ± 3.47 ng/mL in MRL/lpr mice aged 24 weeks. By calculating the molecular weights of anti-dsDNA IgG (150 kDa) to ALW peptide (1.4 kDa), the ALW concentration of 100 μg/mL is sufficient to maintain a high molar ratio (much higher than 5 times of anti-dsDNA IgG). In fact, the maximum solubility of PLP peptide is also 100 μg/mL, less than ALW peptide. Urine was collected every 2 weeks. Six weeks later, the mice were euthanized. Blood, urine, and kidneys were harvested for further analysis.

The ALW and PLP peptides were also intravenously injected into BALB/c mice with the same dosages, frequencies, and treatment period. There were no detectable anti-dsDNA antibodies in sera after such treatment (data not shown). All animal protocols were approved by the Hospital Research Ethics Committee.

### Enzyme-linked immunosorbent assays (ELISAs)

ELISAs were performed with serum or urine according to the manufacturer’s instructions. Immunoassay kits (targeting mouse TGF-β1 and platelet-derived growth factor B [PDGF-B]) were purchased from R&D Systems, and the connective tissue growth factor (CTGF) kit was from MyBiosource Inc. (San Diego, CA). Mouse microalbuminuria kit was purchased from Elabscience (China). Total anti-dsDNA IgG and IgG isotype concentrations were measured by ELISA in serum diluted 1:200 as described [17]. The optical density values were read at 450 nm.

### Measurement of biochemical parameters and lymphadenopathy

Both urea nitrogen and creatinine were determined in urine or serum samples according to the manufacturer’s instructions (BioAssay Systems). In addition, a lymphadenopathy score was assigned using a scale of 0 to 4, with 0 being none; 1, a single palpable lymph node anywhere; 2, bilateral axillary, femoral, or cervical nodes; 3, generalized palpable femoral, axillary, and cervical nodes; and 4, massive generalized adenopathy as described previously [18].

### Immunohistochemistry and histological evaluation

After deparaffinization and rehydration, paraffin sections were blocked with Dual Endogenous Enzyme Block (DAKO, Glostrup, Denmark) for immunohistochemistry. The primary antibody was rabbit anti-CD3 (or B220, Iba-1, Ki-67, TGF-β1, PDGF-B, CTGF, α-smooth muscle actin [SMA], fibronectin, collagen I) IgG (Abcam, Cambridge, MA). The secondary antibody was polymer-horseradish peroxidase-labeled goat anti-rabbit IgG (DAKO). The detection of glomerular IgG deposition was performed with horseradish peroxidase-labeled goat anti-mouse IgM, IgG, IgG1, IgG2a, IgG2b, and IgG3 (Thermo Fisher Scientific) as described previously [17]. Finally, sections were incubated with 3, 3′-diaminobenzine-chromogen substrate (DAKO) and counterstained with hematoxylin. The stains were scored by a renal pathologist blinded to the mice grouping.

To determine renal histopathology, 3-μm-thick paraffin sections of the kidney were stained with hematoxylin & eosin (H&E), periodic acid Schiff (PAS), and Masson in that order. Renal histology was assessed blindly by an experienced pathologist. Renal histopathological changes were quantitated as described previously [18]. The scores of mesangial proliferation and PAS+ deposition were 0 to 4 (0, absent; 1, mild; 2, mild-moderate; 3, moderate; 4, severe). The grades of crescents and tubular lesions (atrophy, casts, dilatation, inflammatory infiltrates) were each scored from 0 to 4 (0, absent; 1, in < 25% of the section; 2, in 25–50% of the section; 3, in 50–75% of the section, and 4, in > 75% of the section). The maximum score for each mouse was 16 (mesangial hyperplasia, PAS+ deposits, crescent, and tubulopathy were 4 points, respectively).

### Transmission electron microscopy (TEM) and immunofluorescence

Kidney tissue (1 mm × 1 mm) was fixed in 2% glutaraldehyde and 4% formaldehyde in 0.1 M phosphate-buffered saline (PBS; pH 7.2) overnight at 4 °C and further fixed in 1% osmium tetroxide in s-collidine buffer. Sections of 0.1 μm were cut, stained with lead citrate and uranyl acetate, and examined at 80 kV with a JEOL 1200EX electron microscope (JEOL, Peabody, MA) [12].

Immunofluorescent detection was performed with frozen sections by O.C.T. (Sakura Finetek, Torrance, CA). After the slides were fixed in precooled acetone, they were air dried for 30 min and then blocked with 10% bovine serum albumin (BSA) in PBS, followed by incubation with Alexa Fluor® 488-conjugated goat anti-mouse IgM, IgG, IgG1, IgG2a, IgG2b, and IgG3 (Abcam, Cambridge, MA) for 1 h at 37 °C. Then, sections were counterstained with 4′,6-diamidino-2-phenylindole and observed under digital confocal microscopy (Leica, Wetzlar, Germany). Ten random glomeruli in each section were measured for fluorescence intensity of IgG subclass expressions.

### Quantitative real-time polymerase chain reaction (qRT-PCR)

Total RNA was extracted from fresh tissue by Trizol reagent (Ambion, Carlsbad, CA). Complementary DNA was generated with a commercial kit (Takara Bio, Kyoto, Japan). qRT-PCR was performed in triplicate with TB green stain (Takara Bio) and the ABI PRISM 7900HT Sequence Detection System (Applied Biosystems, Waltham, MA). The PCR primers (Sangon Biotech) are listed in Additional file 1: Table S1. The expression levels of the objective genes were calculated with the 2^−ΔCt^ method.

### Western blotting

The protein lysates were prepared from fresh kidney tissue. Samples were separated on electrophoresis gels and then transferred onto polyvinylidene difluoride membrane (Millipore, Billerica, MA). The primary antibodies were rabbit antibodies to α-SMA, fibronectin, or collagen I (Abcam), and rabbit antibodies to glyceraldehyde-3-phosphate dehydrogenase (Cell Signaling Technology). The secondary antibody was horseradish peroxidase-conjugated goat anti-rabbit IgG (Proteintech Group). The signal was measured by an enhanced chemiluminescence kit (Millipore). The intensities of blot bands were quantitated by ImageJ software and normalized to glyceraldehyde-3-phosphate dehydrogenase values.

### Statistical analysis

All data were expressed as mean ± standard error of mean. GraphPad Prism version 6.0 (GraphPad Software, La Jolla, CA) was used for statistical analysis. Analysis of variance (ANOVA) was used for the comparison of more than two groups. Then, the differences between two groups were compared by the two-tailed Student *t* test. Differences were considered to be statistically significant at *p* < 0.05.

## Results

### ALW peptide shows high renal exposure

After intravenous administration of 12.5 mg/kg ALW or PLP peptide to CD1 mice, the elimination half-life (*T*_1/2_) of ALW peptide in plasma was 0.315 h (Additional file 1: Table S2), whereas the *T*_1/2_ of PLP peptide was 9.92 h (Additional file 1: Table S3). The mean residence time (MRT_0-inf_) of ALW (PLP) was 0.130 h (0.227 h), and the plasma clearance rate was 789 mL/min/kg (14.7 mL/min/kg). Furthermore, ALW exposure in plasma was significantly higher than that in liver, lung, and kidney tissues. Renal tissue exposure was the highest among these three tissues, approximately 11.2% of plasma exposure (Additional file 1: Table S4), followed by the lung and liver, which were 1.99% and 0.892%, respectively (Additional file 1: Table S5–S6). This suggested that ALW peptide was mainly metabolized by the kidney tissue.

### ALW peptide decreases glomerular deposition of IgG in MRL/lpr mice

**Fig. 1:**
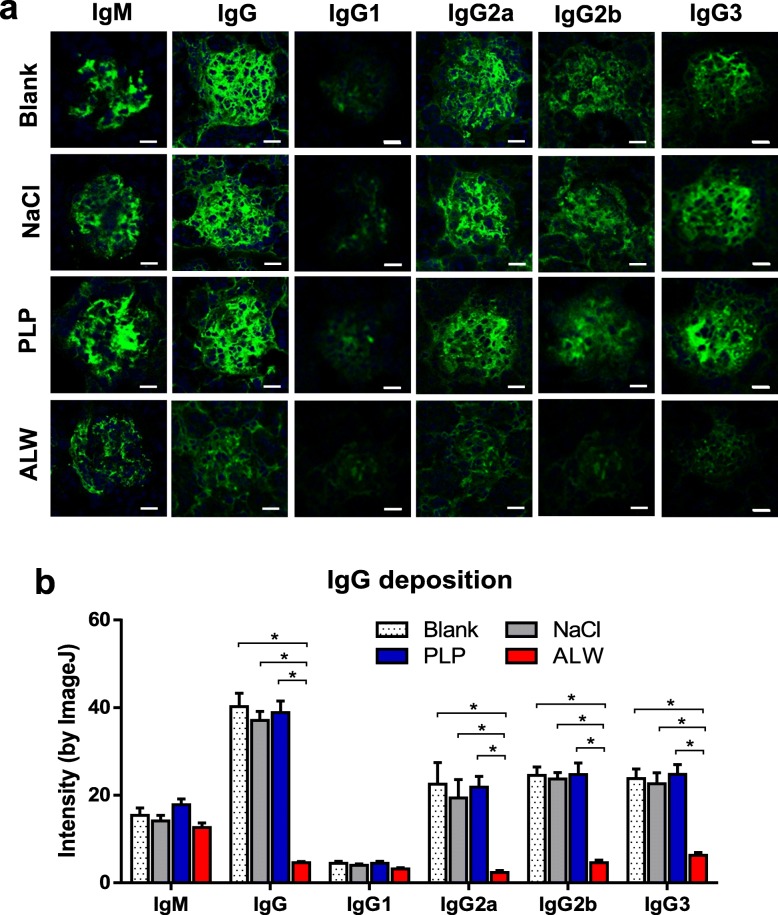
Glomerular IgG deposition is decreased in ALW-treated MRL/lpr mice. **a** Representative images are shown for glomerular IgG subclasses by immunofluorescence. **b** Fluorescence intensities of IgG deposition were quantitated by ImageJ, showing significant decreases of IgG, IgG2a, IgG2b, and IgG3 deposition in glomeruli of ALW-treated mice. There is no difference in the glomerular deposition of IgM and IgG1 between each group. Scale bar = 50 μm. *p < 0.001

**Fig. 2:**
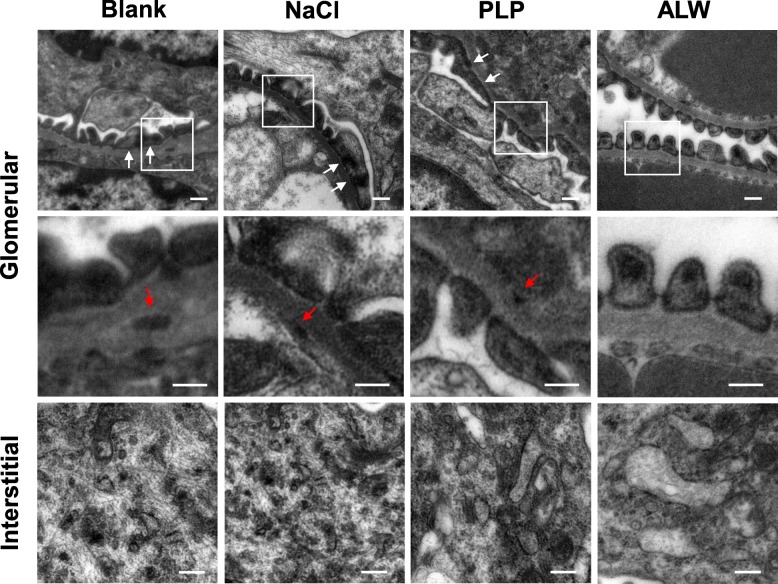
Transmission electron microscopy confirms ultrastructural changes in glomeruli and tubulointerstitium. Less patchy effacement of podocyte foot processes (white arrow) and electron dense deposits are observed along the glomerular base membrane (red arrow) in the mice of the ALW group. Accordingly, intracellular swelling and electron dense deposits decreased in the tubulointerstitial areas. Representative images are shown. Scale bar = 2 μm

Deposition of antibodies plays a pivotal role in the pathogenesis of LN. We found significantly decreased deposition of IgG, IgG2a, IgG2b, and IgG3 in the glomeruli of ALW-treated mice by immunofluorescence (Fig. 1a, b). Among these subclasses of IgG, the fluorescence intensity of IgG3 was the strongest, followed by IgG2a and IgG2b. However, there was no significant difference in the glomerular deposition of IgM between every single group. Moreover, the fluorescence intensity of IgG1 was so weak that the difference between groups was not significant. By means of immunohistochemistry, we re-validated that the glomerular deposition of IgG decreased in ALW-treated mice (Additional file 2: Figure S1). IgM staining was weakly positive by immunohistochemistry, and no significant difference was found between groups. In addition, we detected no positive glomerular staining of IgG1, IgG2a, IgG2b, and IgG3 in all mice through immunohistochemistry. Moreover, TEM results also demonstrated fewer electron dense deposits along the glomerular basement membrane and less fusion of podocyte foot process or tubulointerstitial damage in ALW-treated mice (Fig. 2), mirroring that ALW peptide decreased glomerular deposition of immune complex in MRL/lpr mice. However, we found no significant differences in anti-dsDNA IgM, IgG, IgG1, IgG2a, IgG2b, and IgG3 titers in sera from all the mice (Additional file 2: Figure S2).

### ALW peptide ameliorates glomerular, tubular, and interstitial injuries in MRL/lpr mice

**Fig. 3:**
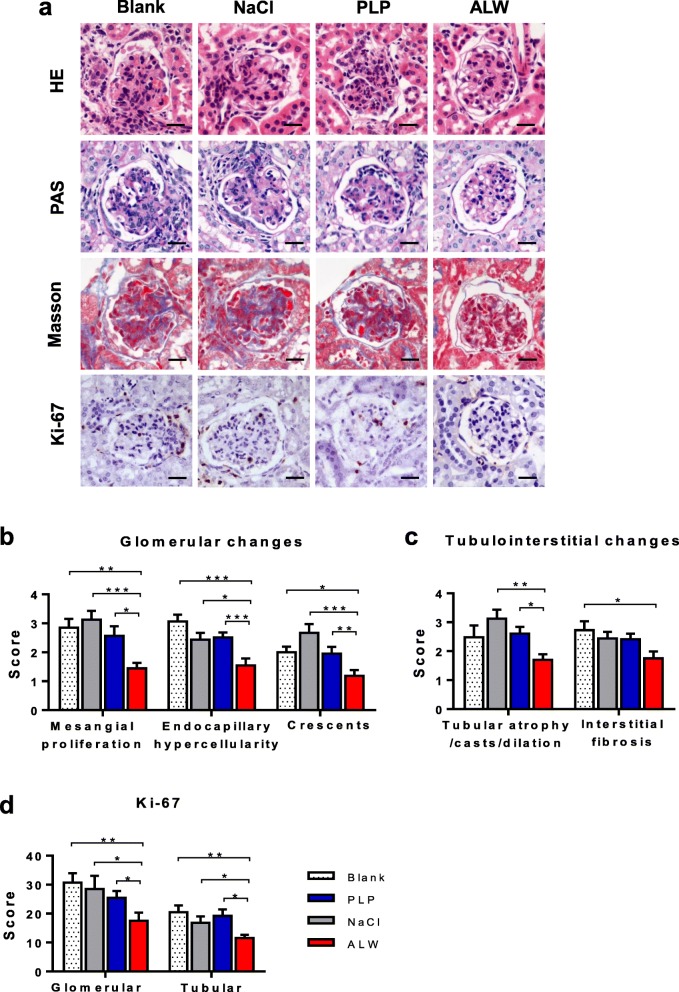
Renal histopathologies are attenuated in ALW-treated MRL/lpr mice. **a** Representative kidney sections (stained with H&E, PAS, Masson, and Ki-67), demonstrating decreased glomerular damage in ALW-treated MRL/lpr mice. **b** Semiquantitative scored results according to H&E and PAS staining showed significantly decreased glomerular changes (mesangial proliferation, endocapillary hypercellularity, and crescents) in the kidney of ALW-treated mice. **c** Semiquantitative scoring results due to H&E, PAS, and Masson staining showed a significant difference in tubular injury of ALW-treated mice compared with the NaCl and blank groups, and in interstitial fibrosis between the ALW and blank groups. **d** Ki-67-stained sections were scored by ImageJ, showing fewer Ki-67-positive cells in both glomerular and tubular areas of ALW-treated mice. Scale bar = 50 μm. *p < 0.05, **p < 0.01, ***p < 0.001

To observe the effect of ALW peptide on renal histopathology, we performed H&E, PAS, and Masson staining of renal sections. ALW-treated mice showed less glomerular injury, including mesangial proliferation, endocapillary hypercellularity, and crescents (Fig. 3a, b). Moreover, Masson staining, along with H&E and PAS staining, showed that tubular injury was decreased in ALW-treated mice compared with that of the NaCl and PLP groups, reflected by less tubular atrophy and dilation and fewer casts (Fig. 3a, c; Additional file 2: Figure S3). Accordingly, Ki-67 staining demonstrated decreased cellular proliferation in both glomerular and tubular areas in the mice of the ALW group (Fig. 3a, d; Additional file 2: Figure S3).

Furthermore, we monitored proteinuria, urea nitrogen, and creatinine every 2 weeks. However, proteinuria was undetectable in all mice during the 2- to 4-week treatment (data not shown). Mice were sacrificed after 6-week treatment. At this time point, urine microalbumin appeared but at low levels. Moreover, there were no differences between these groups. The serum or urine levels of urea nitrogen and creatinine as well as lymph node scores were comparable between these four groups before (data not shown) or after 6-week treatment. See Additional file 2: Figure S4.

### Infiltration of inflammatory cells decreases in the kidneys of ALW-treated mice

**Fig. 4:**
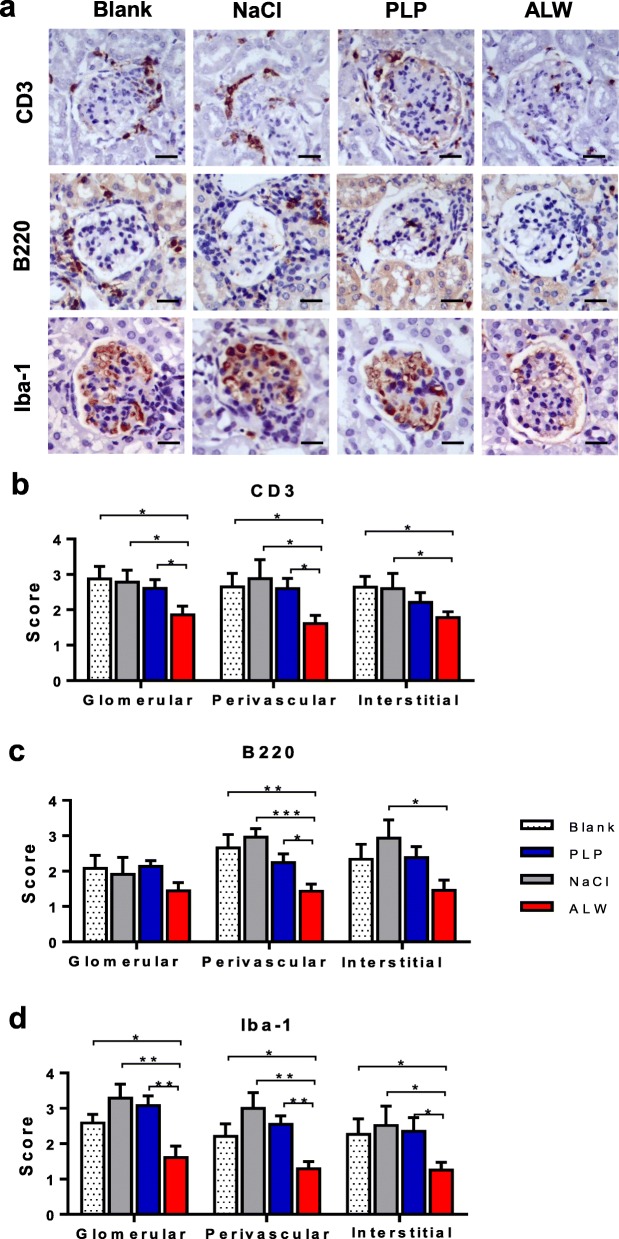
Inflammatory cell infiltration was alleviated in the kidneys of ALW-treated MRL/lpr mice. **a** Representative images of CD3, B220, and Iba-1 staining are shown. **b** Semiquantitative scores of CD3 were determined separately in the glomerular, perivascular, and interstitial areas, showing a significant decrease in these areas of ALW-treated mice. **c** Sections of B220 staining were scored similarly, demonstrating a marked decrease in the perivascular regions of ALW-treated mice, but not in the glomerular and interstitial areas. **d** Semiquantitative scores of Iba-1 showed less infiltration of macrophages in the glomerular, perivascular, and interstitial areas in the mice of the ALW group. Scale bar = 50 μm. *p < 0.05, **p < 0.01, ***p < 0.001

The infiltration of inflammatory cells contributes to the development of LN, including T cells, B cells, and macrophages. By immunohistochemistry, we tested the infiltration of these cells in kidney tissue of the four groups. The CD3 (T cells), B220 (B cells), or Iba-1 (activated macrophages) marker was applied accordingly. Our results showed that CD3-positive cells decreased distinctly in glomerular and perivascular areas in the mice of the ALW group (Fig. 4a, b). Compared with the NaCl and blank groups, the ALW group had less interstitial infiltration of CD3-positive cells (Fig. 4a, b). Moreover, the perivascular infiltration of B220-positive cells was significantly decreased in the kidney tissue of ALW-treated mice (Fig. 4). Furthermore, the glomerular, perivascular, and interstitial infiltration of Iba-1-positive cells were all markedly alleviated in the mice of the ALW group (Fig. 4a, d). Interstitial infiltration of inflammatory cells is also presented in Additional file 2: Figure S3.

### Profibrotic cytokines are less expressed in both sera and kidneys of ALW-treated mice

**Fig. 5:**
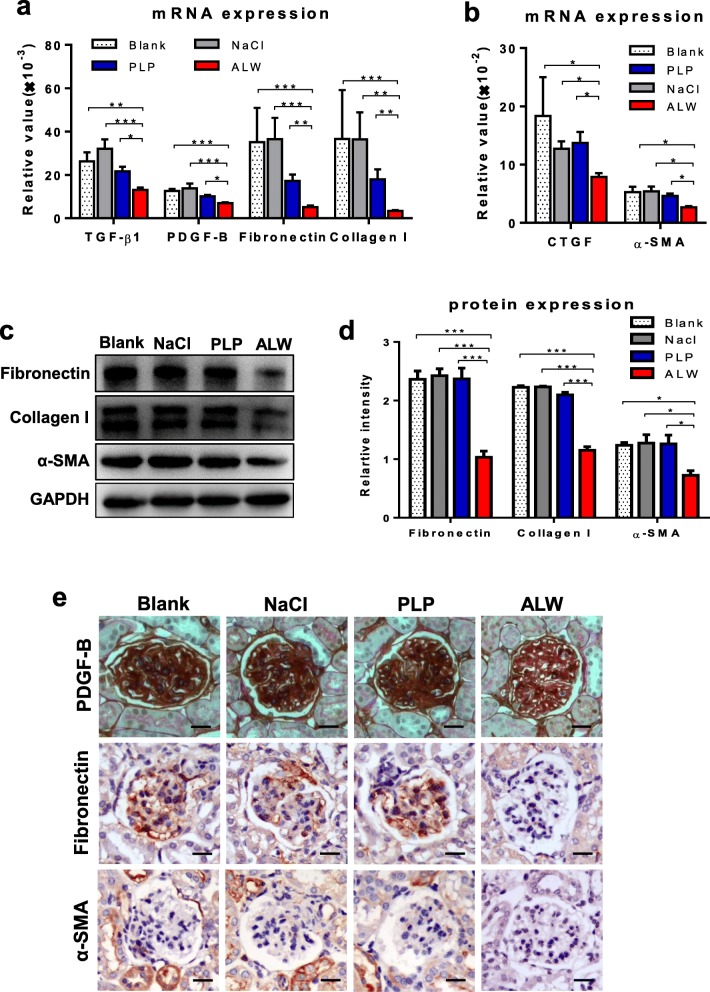
ALW peptide suppresses the production of profibrotic cytokines in kidney of MRL/lpr mice. **a** and **b** The mRNA expression levels of TGF-β1, PDGF-B, CTGF, α-SMA, fibronectin, and collagen I were determined in tissue lysates. **c** and **d** By western blotting, α-SMA, fibronectin, and collagen I were detected in the protein lysates of kidney. **e** By immunohistochemistry, the sections were detected for PDGF-B, fibronectin, and α-SMA. Scale bar = 50 μm. *p < 0.05, **p < 0.01, ***p < 0.001

Fibrosis is one of the main pathological mechanisms in progressive LN and plays an important role in the development of end-stage renal disease which is mainly manifested in increased synthesis of extracellular matrix and the presence of glomerulosclerosis. In our study, we found that ALW-treated MRL/lpr mice had lower mRNA expression levels of TGF-β1, PDGF-B, and CTGF in kidney compared with the other three groups (Fig. 5a, b). The expressions of TGF-β1 and PDGF-B were further reflected by immunohistochemical staining of renal sections (Fig. 5e and Additional file 2: Figure S5). At the same time, the serum levels of these cytokines in ALW group mice were all significantly decreased (Additional file 2: Figure S6). Moreover, the serum levels of TGF-β1, PDGF-B, and CTGF correlated positively with renal chronicity index (Additional file 2: Figure S6).

In addition, the mRNA expression levels of the extracellular matrix, such as α-SMA, fibronectin, and collagen I, were evidently decreased in the kidney tissue of the ALW-treated mice (Fig. 5a, b). Their protein levels were detected in tissue lysates by Western blot analysis, showing lower expressions in ALW-treated mice (Fig. 5c, d). Moreover, the results of immunohistochemical staining were consistent with those discussed previously (Fig. 5e and Additional file 2: Figure S5), suggesting that ALW peptide decreased the expressions of extracellular matrix and blocked the progression of renal fibrosis.

## Discussion

This study shows that ALW peptide ameliorates renal injury in LN of MRL/lpr mice. First, we examined the pharmacokinetic and biodistribution of ALW peptide in vivo, which showed quick plasma clearance and high renal exposure. Nevertheless, ALW peptide decreased glomerular deposition of IgG in MRL/lpr mice, accompanied by markedly attenuated histopathologies, such as glomerular proliferation and infiltration of inflammatory cells. Furthermore, profibrotic cytokines, including TGF-β1, PDGF-B, and CTGF, were also inhibited in the serum and kidney of ALW-treated MRL/lpr mice, along with decreased expressions of extracellular matrix in kidney tissue.

Given the key role of anti-dsDNA antibodies in the pathogenesis of LN, development of specific inhibitors of anti-dsDNA antibodies may be a good method to manage LN. Several polypeptides, specifically reacting with mouse anti-dsDNA IgG2b, were identified by the peptide display phage library. For example, administration of D-form DWEYS peptide protected SCID mice from the renal deposition of IgG2b [15]. FISLE-412, a small peptidomimetic modified from DWEYS peptide, blocked the binding of DNA to monoclonal mouse and human anti-dsDNA antibodies with improved affinity and selectivity [15]. In addition, intravenous administration of pCons to NZB×NZW F1 lupus mice once a month, a 15-mer peptide (FIEWNKLRFRQGLEW) based on the determinants of MHC class I and class II T cell in the V_H_ region of murine anti-dsDNA IgG2a or IgG2b, induced immune tolerance for anti-DNA antibodies in autoreactive T cell helper populations and delayed the emergence of multiple autoantibodies [19, 20]. Our results were consistent with these previous studies that peptide can mimic the antigenicity of DNA and block the combination of anti-dsDNA antibodies to autoantigens by competing with DNA for binding sites. What is worth pointing out is that the remaining peptides described above were selected by a single isotype, whereas ALW peptide bound to all four IgG isotypes in mouse and human, which will inhibit the pathogenic polyclonal anti-dsDNA response more extensively.

The ALW peptide is a small molecule with a molecular weight of 1.4 kDa. It is easy to dissolve in water or PBS and can be injected intravenously. Because there is no methionine, cysteine, and glutamine, which are related with the oxidation, cyclization, and degradation of peptides, ALW may have greater physiological stability [21]. In addition, the ALW peptide has a relatively neutral net charge (pI = 7.38), which probably decrease unnecessary, nonspecific protein-protein interactions. However, ALW peptide would be catalyzed weakly by anti-dsDNA IgGs [22]. Here, after chemical modification, the resistance of ALW peptide to catalysis was enhanced without affecting its biological activity by substituting two terminal residues of A (alanine) and P (proline) with D-form ones, respectively [23]. With regard to metabolism and distribution in vivo, ALW peptide showed quick elimination, with a *T*_1/2_ of 0.315 h. Furthermore, renal tissue exposure of ALW peptide was much higher than that of the liver and lung, suggesting that ALW peptide was mainly located in and metabolized by the kidney.

Potential immunogenicity is a concern about the systemic administration of peptide. In fact, subcutaneous immunization of DNA-mimicking peptides in adjuvant induces renal IgG deposition in non-autoimmune mice [24, 25]. However, subcutaneous immunization of DNA-mimicking peptide alone induces no such autoantibodies [25]. It is widely acceptable that intravenous injection is associated with much less immunogenicity as compared with subcutaneous immunization [26–28]. In this study, serum anti-dsDNA antibodies are comparable between the ALW and control groups, indicating no effect of ALW injection on antibody production in MRL/lpr mice. Moreover, intravenous injection of ALW peptide induced no anti-dsDNA antibodies in BALB/c mice. Therefore, intravenous injection of ALW is not expected to be immunogenic, especially without adjuvant.

Different antibodies had differential pathogenic potential, which is related to the affinity and specificity to renal antigens, homology of the idiotype, and the nature and location of specific amino acid residues in complementarity determining regions (CDR) [13, 29]. We have previously demonstrated that the constant region contributed to the antigenic specificity and renal pathogenicity of murine anti-dsDNA antibodies [12]. The relative affinity of PL9-11 anti-DNA antibodies for dsDNA was IgG3 > IgG2a > IgG1 > IgG2b = IgM [12]. In lupus-prone mice, IgG2a, IgG2b, and IgG3 are the relatively richest in glomeruli of active LN [30]. Moreover, the affinity of IgG2a and IgG3 to glomerular and mesangial cells was the highest [12]. In our previous studies, the ALW peptide has differential affinity to anti-dsDNA antibodies in the order of IgG2b > IgG2a > IgG3 > IgG1, whereas PLP peptide had no significant binding to IgG2a and IgG3. Here, we detected markedly decreased deposition of IgG, IgG2a, IgG2b, and IgG3 in the glomeruli of ALW-treated MRL/lpr mice by immunofluorescence. However, there was no significant difference in the glomerular deposition of IgM between every single group, suggesting weak affinity of ALW peptide to IgM. Among these subclasses, IgG3 has the strongest fluorescence intensity, followed by IgG2a and IgG2b. The fluorescence intensity of IgG1 was weak, and there was no significant difference between groups, which agreed with our previous findings.

Renal inflammatory cell infiltration is crucial in the development of nephritis [31]. T cells, B cells, and macrophages are known to promote renal injury in MRL/lpr mice [32–34]. Abnormal activated T cell in LN amplified inflammation by secreting pro-inflammatory cytokines, helped B cells to produce autoantibodies, and recruited autoreactive memory T cells [35, 36]. Simultaneously, aberrant B cell subsets promoted the production of autoantibodies and presentation of autoantigens to effector cells [37, 38]. In our study, we found that perivascular infiltration of T and B cells decreased significantly in the mice of the ALW group. Moreover, infiltration of macrophage cells was decreased in the glomerular, perivascular, and interstitial areas of the kidneys in ALW-treated mice, reflecting the remission of inflammatory infiltration.

In our study, we detected no significant difference in the microalbuminuria and serum urea nitrogen or creatinine. This might be due to the young ages (15 weeks old after treatment) of these mice which had only mild glomerulonephritis. Actually, female MRL/lpr mice at age of 12 weeks develop proteinuria under 30 mg/dl and 300 to 1000 mg/dl after 20 weeks [39]. Since frequent injection induced vascular deformation or fibrosis in the tails, the experiments ceased after 6-week treatment. Hence, the differences in proteinuria might be observed upon prolongation of the administration interval and treatment period. In future study, more stable ALW peptide will afford such expectation.

Renal fibrosis is one of the final outcomes of LN. Anti-dsDNA antibodies induce tubular epithelial myofibroblast transdifferentiation and promote the expression of profibrotic factors [40]. Our results showed that renal mRNA expression levels of fibrotic markers, such as TGF-β1, PDGF-B, CTGF, α-SMA, fibronectin, and collagen I, were significantly decreased in ALW-treated mice, mirrored by immunohistochemical results of kidney sections except for CTGF. In addition, the serum levels of TGF-β1, PDGF-B, and CTGF in the mice of the ALW group were all evidently decreased. Furthermore, the protein levels of α-SMA, fibronectin, and collagen I by Western blot assay decreased markedly in ALW-treated mice compared with the other groups, consistent with the results discussed above.

## Conclusions

**Fig. 6:**
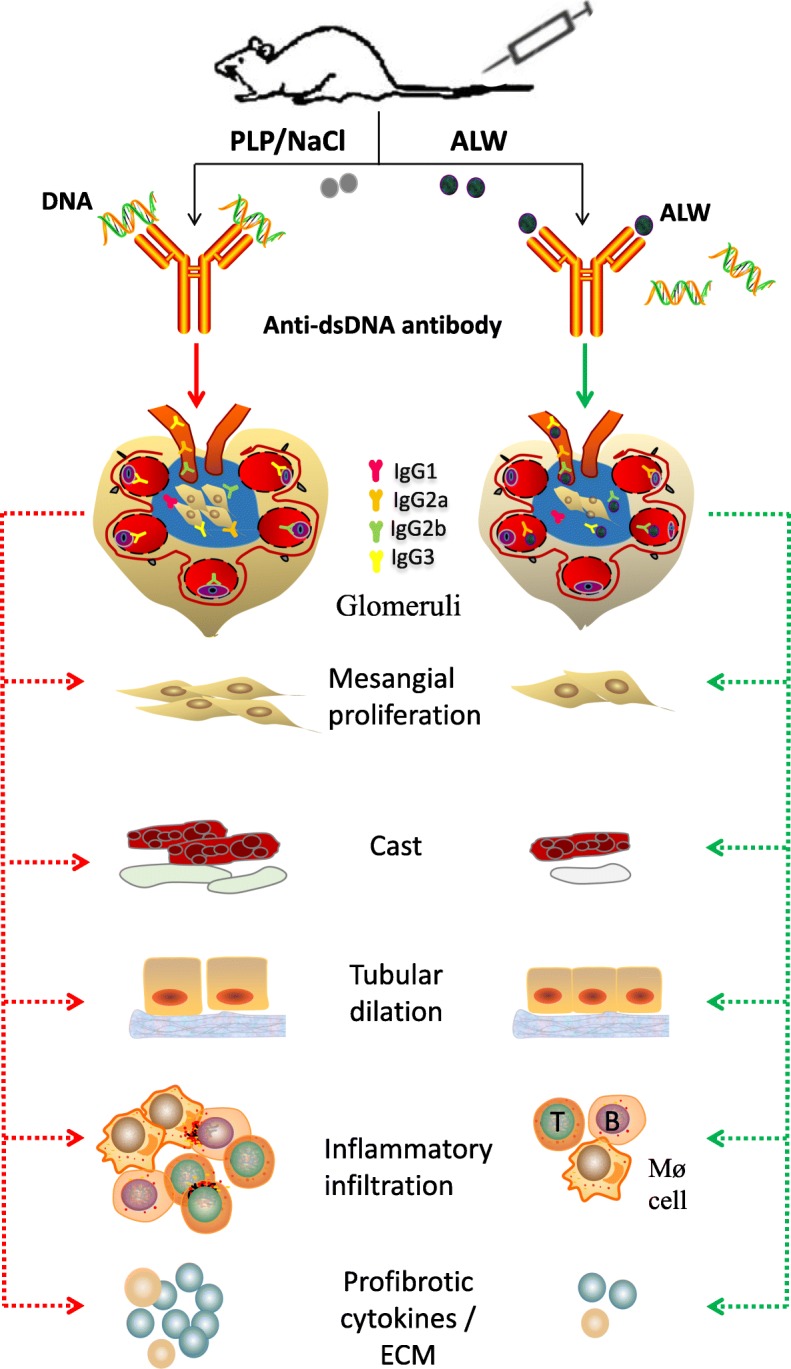
Overview of the effect of ALW peptide on the progress of LN in MRL/lpr mice. After the administration of ALW peptide to MRL/lpr mice, the deposition of IgG, IgG2a, IgG2b, and IgG3 in glomeruli and mesangial cells are significantly reduced, following by alleviated glomerular injury, including mesangial proliferation, endocapillary hypercellularity, and crescents and tubular injury reflected by less tubular atrophy and dilation and fewer casts. At the same time, the infiltration of T cell, B cell, and macrophage cell is inhibited in the kidney of the ALW-treated mice. Renal and serum TGF-β1, PDGF-B, and CTGF accompanied by extracellular matrix (ECM) in the kidney are all declined in ALW-treated mice

Based on our findings, we indicate that ALW peptide attenuates renal injuries in LN of MRL/lpr mice (Fig. 6). A limitation in this study was that we performed only high-dose administration of ALW peptide to a murine model of LN. The main purpose of this study was to discuss preliminarily the effect of the ALW peptide on the onset and progress of LN. The most important and troublesome technical problem is to improve the stability of peptides in vivo through different chemical modification without affecting their biological characteristics. In the near future, we plan to design and screen out more stable ALW peptide and prolong the interval of administration and period of treatment. Besides, we will administrate ALW peptide with different doses to lupus-prone mice to further explore the effect of ALW peptide on the evolvement of LN, survival rate of mice, immune balance such as T cell and B cell subsets, and probe into the relationship between drug dose and disease activity. Nevertheless, our results highly confirmed that ALW peptide suppressed the progression of LN in MRL/lpr mice, providing novel options for the management of patients with LN.

## Supplementary information

**Additional file 1: TableS1.** Primer sets used in PCR. **TableS2.** The pharmacokinetic parameters of ALW peptide in plasma. **Table S3.** The pharmacokinetic parameters of PLP peptide in plasma. **Table S4.** The pharmacokinetic parameters of ALW peptide in kidney. **Table S5.** The pharmacokinetic parameters of ALW peptide in liver. **TableS6.** The pharmacokinetic parameters of ALW peptide in lung.

**Additional file 2: Figure S1.** Representative images of antibody staining by immunohistochemistry are shown, which showed markedly decreased IgG deposition in the glomeruli of ALW-treated MRL/lpr mice, but no difference in IgM deposition between groups. Scale bar = 50 μm, **Figure S2.** Serum anti-dsDNA antibodies are not altered in MRL/lpr mice. There were no significant differences in the titers of anti-dsDNA IgM (**a**), anti-dsDNA IgG (**b**), anti-dsDNA IgG1 (**c**), anti-dsDNA IgG2a (**d**), anti-dsDNA IgG2b (**e**), anti-dsDNA IgG3 (**f**) (*p* > 0.05), **Figure S3.** Tubulointerstitial changes are attenuated in ALW-treated MRL/lpr mice. **a** Representative kidney sections (stained with H&E, PAS, Masson, and Ki-67) showed less tubulointerstitial damage in ALW-treated MRL/lpr mice. **b** Representative images of CD3, B220, and Iba-1 staining showed fewer inflammatory cells in ALW-treated MRL/lpr mice. Scale bar = 50 μm, **Figure S4.** Proteinuria, renal function parameters, and lymph node scores are comparable in different groups of MRL/lpr mice. The urine levels of microalbumin (**a**), creatinine (**b**), and urea nitrogen (**c**) were measured after 6-week treatment. The serum levels of creatinine (**d**) and urea nitrogen (**e**) were determined accordingly. At the same time point, lymph nodes were scored in all mice (**f**). There were no significant differences in any parameter between these four groups (*p* > 0.05)., **Figure S5.** Representative images of immunohistochemical staining demonstrated evidently decreased expressions of TGF-β1 and collagen I in kidneys of ALW-treated mice. Scale bar = 50 μm, **Figure S6.** Profibrotic cytokines were attenuated in serum of ALW-treated MRL/lpr mice. Serum of TGF-β1 (**a**), PDGF-B (**b**), and CTGF (**c**) were much lower in the mice of the ALW group. The serum levels of TGF-β1 (**d**), PDGF-B (**e**), and CTGF (**f**) correlated positively with renal chronicity index. *p < 0.05, **p < 0.01, ***p < 0.001.

## Data Availability

The datasets used and/or analyzed during the current study are available from the corresponding author on reasonable request.

## Abbreviations

CTGF: Connective tissue growth factor
ELISA: Enzyme-linked immunosorbent assay
LN: Lupus nephritis
PDGF-B: Platelet-derived growth factor B
qRT-PCR: Quantitative real-time polymerase chain reaction
TEM: Transmission electron microscopy
TGF-β1: Transforming growth factor-beta1
