# Supplementary material for: ALW peptide ameliorates lupus nephritis in MRL/lpr mice
Source: Arthritis Res Ther. 2019 Dec 2;21:261. doi: 10.1186/s13075-019-2038-0 (PMC6889545; doi:10.1186/s13075-019-2038-0)
Supplement: Supplementary file 1 — Additional file 1: TableS1. Primer sets used in PCR. TableS2. The pharmacokinetic parameters of ALW peptide in plasma. Table S3. The pharmacokinetic parameters of PLP peptide in plasma. Table S4. The pharmacokinetic parameters of ALW peptide in kidney. Table S5. The pharmacokinetic parameters of ALW peptide in liver. TableS6. The pharmacokinetic parameters of ALW peptide in lung. [file 13075_2019_2038_MOESM1_ESM.docx]

**Table S1.** Primer sets used in PCR

| **Target gene** | **Primer (5’-3’)** |
| --- | --- |
| Murine α-SMA | F: 5’-CTGACAGAGGCACCACTGAACC-3’ |
|  | R: 5’-CTCCAGAGTCCAGCACAATACCAG-3’ |
| Murine collagen I | F: 5’-TGAACGTGGTGTACAAGGTC-3’ |
|  | R: 5’-CCATCTTTACCAGGAGAACCAT-3’ |
| Murine CTGF | F: 5’-AAAGCAGCTGCAAATACCAATG-3’ |
|  | R: 5’-AAATGTGTCTTCCAGTCGGTAG-3’ |
| Murine fibronectin | F: 5’-CTATAGGATTGGAGACACGTGG-3’ |
|  | R: 5’-CTGAAGCACTTTGTAGAGCATG-3’ |
| Murine PDGF-B | F: 5’-GTCCAGGTGAGAAAGATTGAGA-3’ |
|  | R: 5’-GTCATGGGTGTGCTTAAACTTT-3’ |
| Murine TGF-β1 | F: 5’-CAACAATTCCTGGCGTTACCTTGG-3’ |
|  | R: 5’-TGTATTCCGTCTCCTTGGTTCAGC-3’ |
| Murine GAPDH | F: 5’-CTCATGACCACAGTCCATGC-3’ |
|  | R: 5’-CACATTGGGGGTAGGAACAC-3’ |

α-SMA: alpha-smooth muscle actin; CTGF: connective tissue growth factor; PDGF-B: platelet-derived growth factor; TGF-β1: transforming growth factor-beta 1; GAPDH: glyceraldehyde phosphate dehydrogenase

**Table S2.** The pharmacokinetic parameters of ALW peptide in plasma

| Parameters | Unit | Estimated Value |
| --- | --- | --- |
| Rsq_adjusted |  | 0.63 |
| Number of points |  | 5 |
| C_0_ | ng/mL | 3642 |
| T_1/2_ | h | 0.32 |
| V_ss_ | L/kg | 6.16 |
| Cl | mL/min/kg | 789 |
| T_last_ | h | 1.00 |
| AUC_0-last_ | ng.h/mL | 258 |
| AUC_0-inf_ | ng.h/mL | 264 |
| MRT_0-last_ | h | 0.10 |
| MRT_0-inf_ | h | 0.13 |
| AUC_Extra_ | % | 2.41 |
| AUMC_Extra_ | % | 26.90 |

T_1/2_: half-life; Cl: plasma clearance; AUC: area under the concentration-time curve; MRT: mean residence time; AUMC: area under the moment curve

**Table S3.** The pharmacokinetic parameters of PLP peptide in plasma

| Parameters | | Unit | Estimated Value |
| --- | --- | --- | --- |
| Rsq_adjusted | |  | 0.68 |
| Number of points |  | 5 |  |
| C_0_ | ng/mL | 85385 |  |
| T_1/2_ | h | 9.92 |  |
| V_ss_ | L/kg | 0.20 |  |
| Cl | mL/min/kg | 14.70 |  |
| T_last_ | h | 24 |  |
| AUC_0-last_ | ng*h/mL | 14121 |  |
| AUC_0-inf_ | ng*h/mL | 14136 |  |
| MRT_0-last_ | h | 0.19 |  |
| MRT_0-inf_ | h | 0.23 |  |
| AUC_Extra_ | % | 0.10 |  |
| AUMC_Extra_ | % | 16.10 |  |

T_1/2_: half-life; Cl: plasma clearance; AUC: area under the concentration-time curve; MRT: mean residence time; AUMC: area under the moment curve

**Table S4.** The pharmacokinetic parameters of ALW peptide in kidney

| Parameters | Unit | Estimated Value |
| --- | --- | --- |
| Rsq_adjusted |  | 0.98 |
| T_1/2_ | h | 0.31 |
| T_max_ | h | 0.08 |
| C_max_ | ng/g | 60.00 |
| AUC_0-last_ | ng.h/g | 25.51 |
| AUC_0-inf_ | ng.h/g | 28.80 |
| MRT_0-last_ | h | 0.33 |
| MRT_0-inf_ | h | 0.46 |
| K_p, kidney_ |  | 0.11 |

T_1/2_: half-life; T_max_: peak time; C_max_: maximum blood concentration; AUC: area under the concentration-time curve; MRT: mean residence time

**Table S5.** The pharmacokinetic parameters of ALW peptide in liver

| Parameters | Unit | Estimated Value |
| --- | --- | --- |
| Rsq_adjusted |  | Not available |
| T_1/2_ | h | Not available |
| T_max_ | h | 0.08 |
| C_max_ | ng/g | 11.80 |
| AUC_0-last_ | ng.h/g | 2.29 |
| AUC_0-inf_ | ng.h/g | Not available |
| MRT_0-last_ | h | 0.14 |
| MRT_0-inf_ | h | Not available |
| K_p, liver_ |  | 0.01 |

T_1/2_: half-life; T_max_: peak time; C_max_: maximum blood concentration; AUC: area under the concentration-time curve; MRT: mean residence time

**Table S6.** The pharmacokinetic parameters of ALW peptide in lung

| Parameters | Unit | Estimated Value |
| --- | --- | --- |
| Rsq_adjusted |  | Not available |
| T_1/2_ | h | Not available |
| T_max_ | h | 0.25 |
| C_max_ | ng/g | 7.33 |
| AUC_0-last_ | ng.h/g | 5.10 |
| AUC_0-inf_ | ng.h/g | Not available |
| MRT_0-last_ | h | 0.49 |
| MRT_0-inf_ | h | Not available |
| K_p, lung_ |  | 0.02 |

T_1/2_: half-life; T_max_: peak time; C_max_: maximum blood concentration; AUC: area under the concentration-time curve; MRT: mean residence time
