# Supplementary material for: ALW peptide ameliorates lupus nephritis in MRL/lpr mice
Source: Arthritis Res Ther. 2019 Dec 2;21:261. doi: 10.1186/s13075-019-2038-0 (PMC6889545; doi:10.1186/s13075-019-2038-0)
Supplement: Supplementary file 2 — Additional file 2: Figure S1. Representative images of antibody staining by immunohistochemistry are shown, which showed markedly decreased IgG deposition in the glomeruli of ALW-treated MRL/lpr mice, but no difference in IgM deposition between groups. Scale bar = 50 μm, Figure S2. Serum anti-dsDNA antibodies are not altered in MRL/lpr mice. There were no significant differences in the titers of anti-dsDNA IgM (a), anti-dsDNA IgG (b), anti-dsDNA IgG1 (c), anti-dsDNA IgG2a (d), anti-dsDNA IgG2b (e), anti-dsDNA IgG3 (f) (p > 0.05), Figure S3. Tubulointerstitial changes are attenuated in ALW-treated MRL/lpr mice. a Representative kidney sections (stained with H&E, PAS, Masson, and Ki-67) showed less tubulointerstitial damage in ALW-treated MRL/lpr mice. b Representative images of CD3, B220, and Iba-1 staining showed fewer inflammatory cells in ALW-treated MRL/lpr mice. Scale bar = 50 μm, Figure S4. Proteinuria, renal function parameters, and lymph node scores are comparable in different groups of MRL/lpr mice. The urine levels of microalbumin (a), creatinine (b), and urea nitrogen (c) were measured after 6-week treatment. The serum levels of creatinine (d) and urea nitrogen (e) were determined accordingly. At the same time point, lymph nodes were scored in all mice (f). There were no significant differences in any parameter between these four groups (p > 0.05)., Figure S5. Representative images of immunohistochemical staining demonstrated evidently decreased expressions of TGF-β1 and collagen I in kidneys of ALW-treated mice. Scale bar = 50 μm, Figure S6. Profibrotic cytokines were attenuated in serum of ALW-treated MRL/lpr mice. Serum of TGF-β1 (a), PDGF-B (b), and CTGF (c) were much lower in the mice of the ALW group. The serum levels of TGF-β1 (d), PDGF-B (e), and CTGF (f) correlated positively with renal chronicity index. *p < 0.05, **p < 0.01, ***p < 0.001. [file 13075_2019_2038_MOESM2_ESM.pptx]

## Slide 1
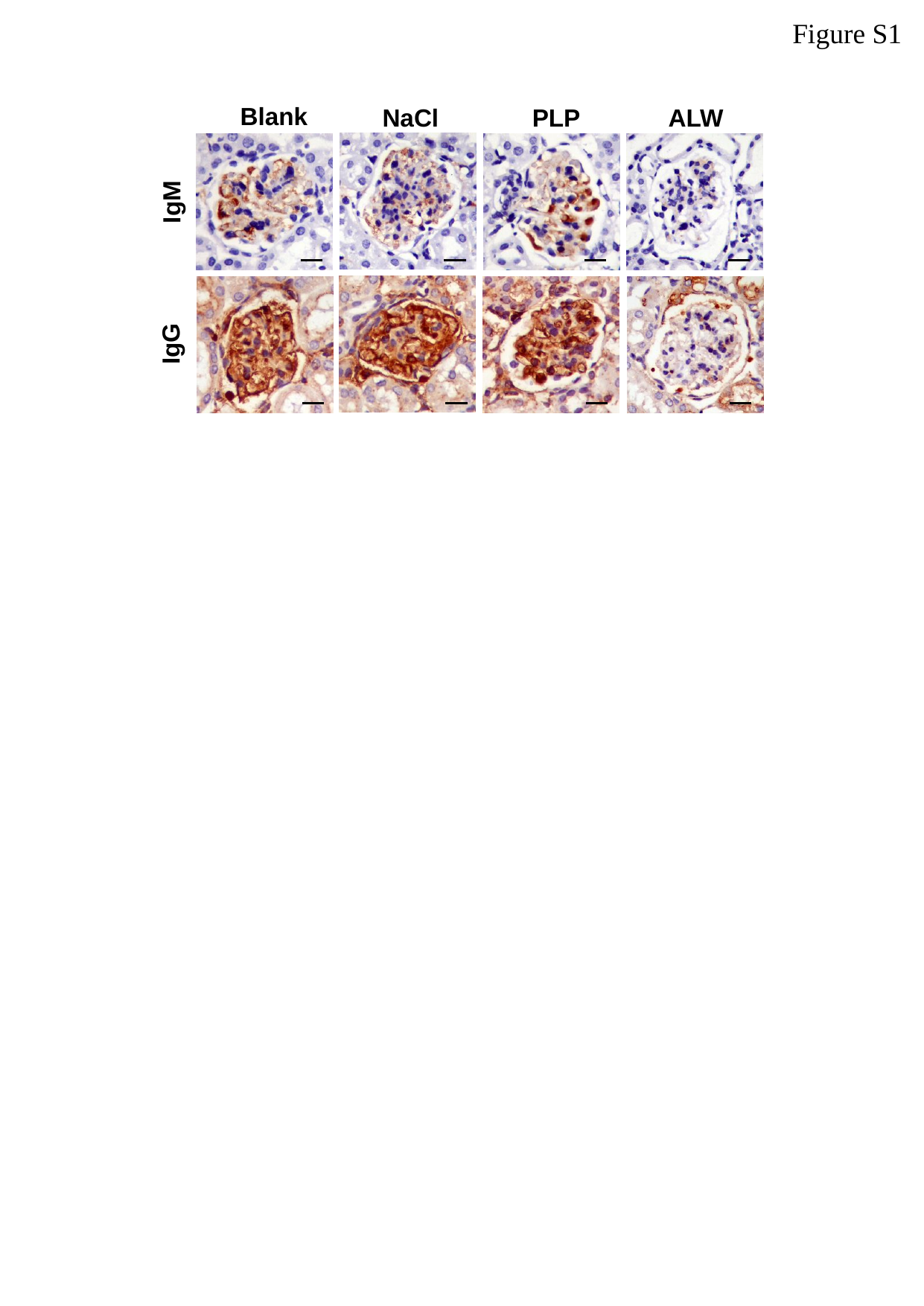

Figure S1
Blank
ALW
NaCl
PLP
IgM
IgG

## Slide 2
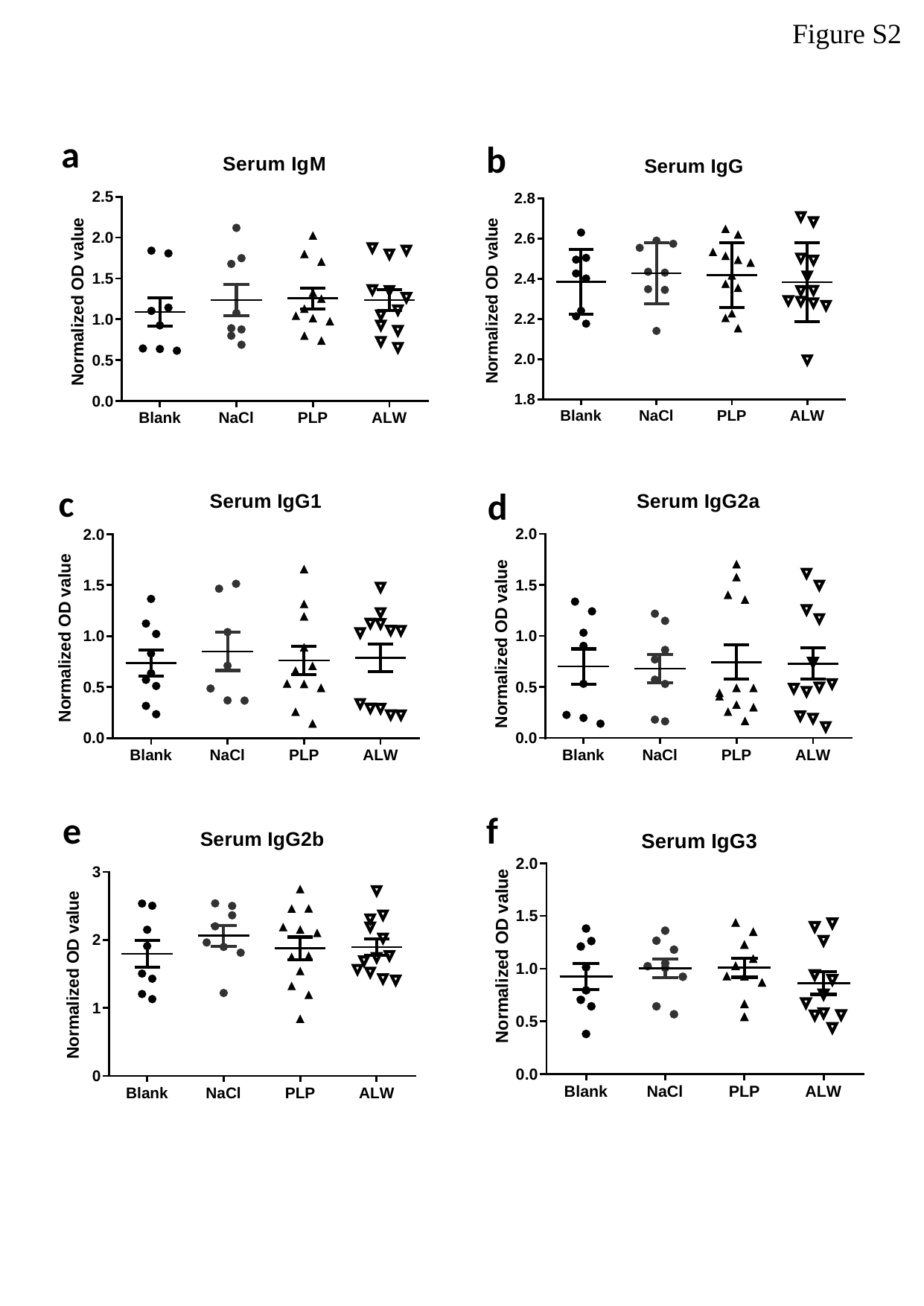

Figure S2
a
b
c
d
e
f

## Slide 3
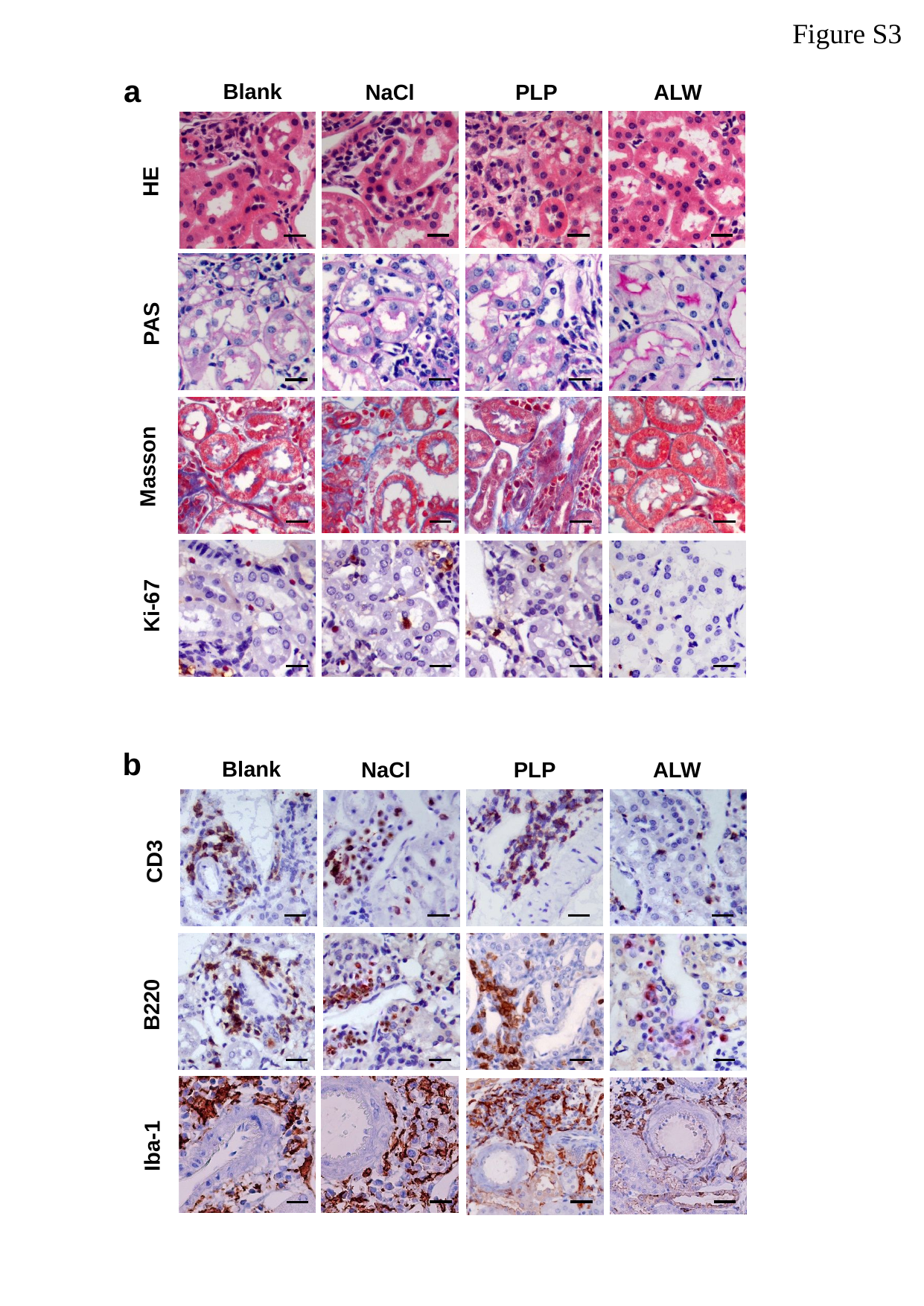

Figure S3
a
Blank
ALW
NaCl
PLP
HE
PAS
Masson
Ki-67
b
Blank
ALW
NaCl
PLP
CD3
B220
Iba-1

## Slide 4
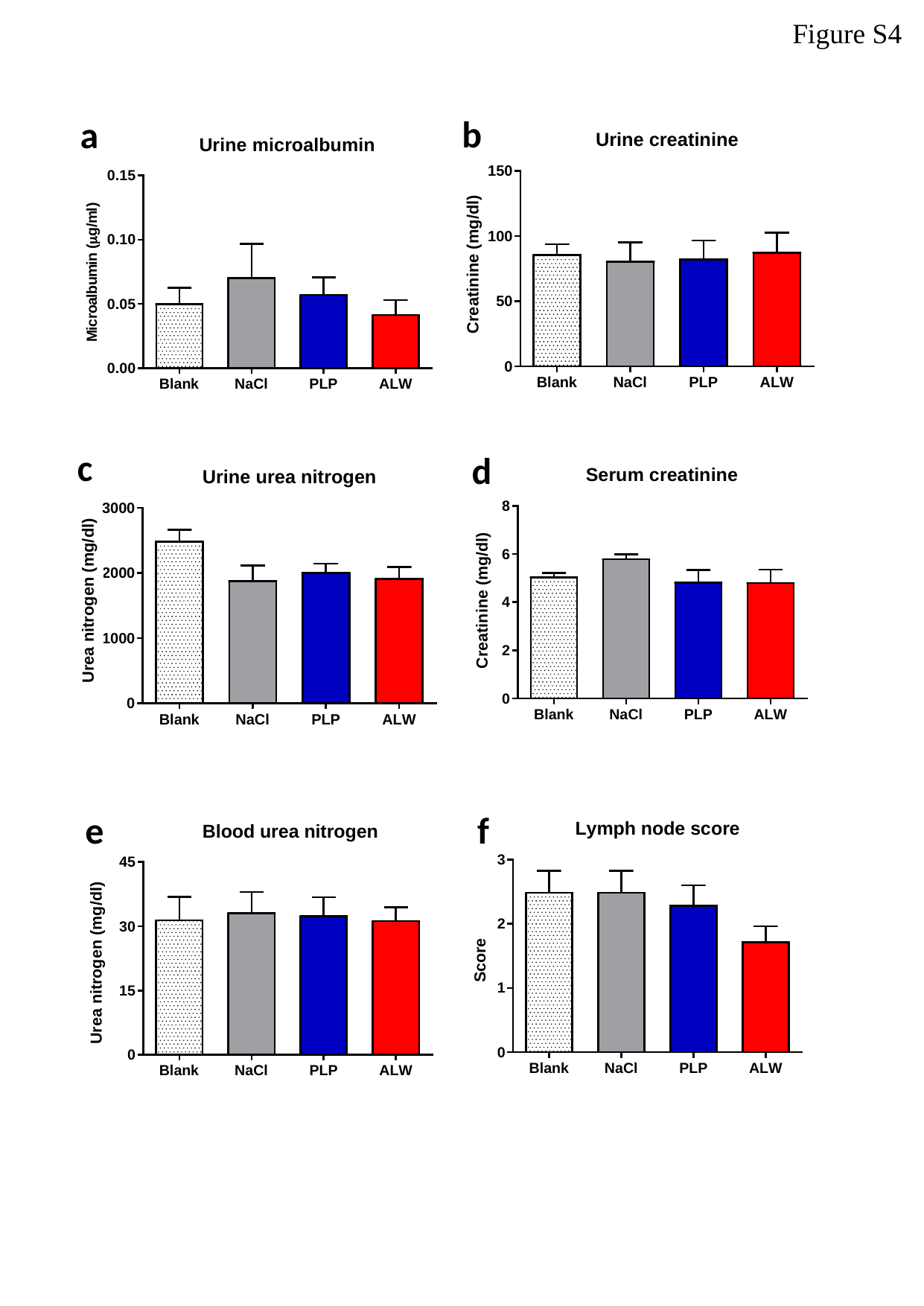

Figure S4
b
a
c
d
f
e

## Slide 5
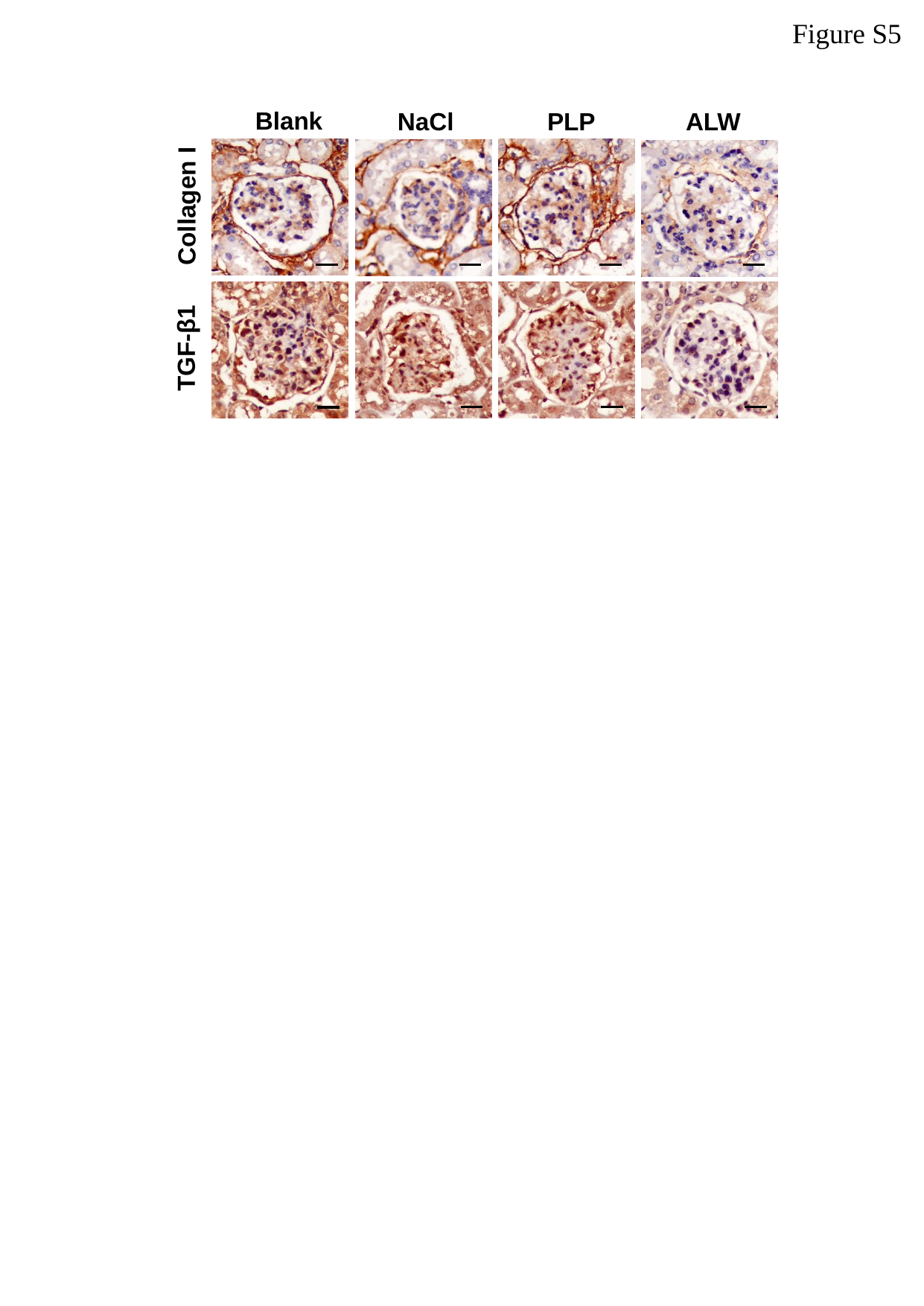

Figure S5
Blank
ALW
NaCl
PLP
Collagen I
TGF-β1

## Slide 6
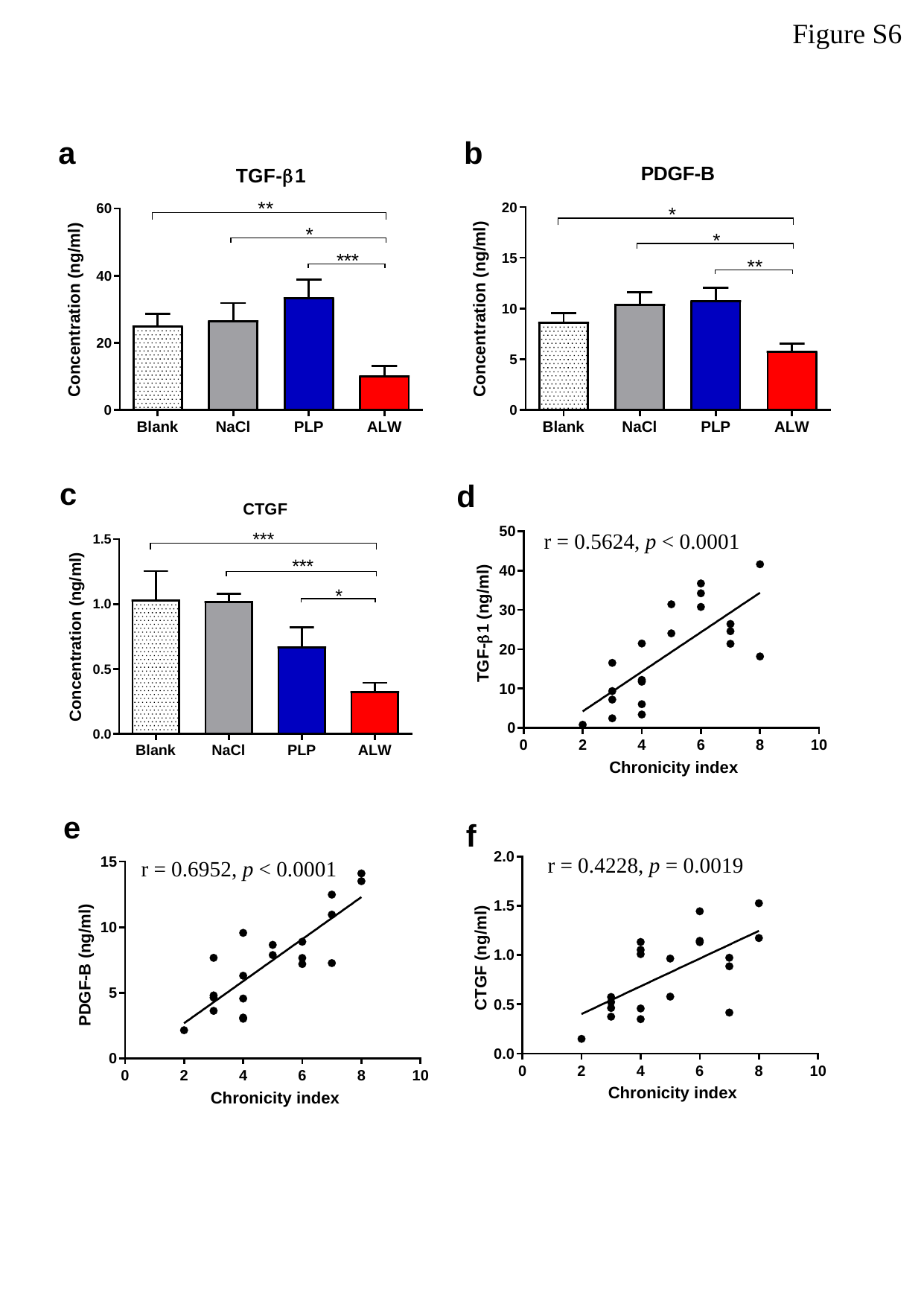

Figure S6
b
a
c
d
r = 0.5624, p < 0.0001
e
f
r = 0.4228, p = 0.0019
r = 0.6952, p < 0.0001
